# Unstable Simple Volatiles and Gas Chromatography-Tandem Mass Spectrometry Analysis of Essential Oil from the Roots Bark of *Oplopanax Horridus* Extracted by Supercritical Fluid Extraction

**DOI:** 10.3390/molecules191219708

**Published:** 2014-11-27

**Authors:** Li Shao, Mei-Hua Bao, Dong-Sheng Ouyang, Chong-Zhi Wang, Chun-Su Yuan, Hong-Hao Zhou, Wei-Hua Huang

**Affiliations:** 1 Department of Human Anatomy, Histology and Embryology, Institute of Neuroscience, Changsha Medical University, Changsha 410219, China; E-Mails: Shaoli82@aliyun.com (L.S.); mhbao78@163.com (M.-H.B.); 2 Department of Pharmacognosy, School of Pharmacy, Changsha Medical University, Changsha 410219, China; 3 Department of Clinical Pharmacology, Xiangya Hospital, Central South University, Changsha 410008, China; E-Mails: Ouyangyj@163.com (D.-S.O.); hhzhou2003@163.com (H.-H.Z.); 4 Hunan Key Laboratory of Pharmacogenetics, Institute of Clinical Pharmacology, Central South University, Changsha 410078, China; 5 Tang Center for Herbal Medicine Research, The Pritzker School of Medicine, University of Chicago, 5841 South Maryland Avenue, MC 4028, Chicago, IL 60637, USA; E-Mails: CWang@dacc.uchicago.edu (C.-Z.W.); CYuan@dacc.uchicago.edu (C.-S.Y.)

**Keywords:** *Oplopanax horridus*, volatile oil, GC/MS, SFE, (*S*,*E*)-nerolidol, *S*-falcarinol

## Abstract

Volatile oil from the root bark of *Oplopanax horridus* is regarded to be responsible for the clinical uses of the title plant as a respiratory stimulant and expectorant. Therefore, a supercritical fluid extraction method was first employed to extract the volatile oil from the roots bark of *O. horridus*, which was subsequently analyzed by GC/MS. Forty-eight volatile compounds were identified by GC/MS analysis, including (*S*,*E*)-nerolidol (52.5%), τ-cadinol (21.6%) and *S*-falcarinol (3.6%). Accordingly, the volatile oil (100 g) was subjected to chromatographic separation and purification. As a result, the three compounds, (*E*)-nerolidol (2 g), τ-cadinol (62 mg) and *S*-falcarinol (21 mg), were isolated and purified from the volatile oil, the structures of which were unambiguously elucidated by detailed spectroscopic analysis including 1D- and 2D-NMR techniques.

## 1. Introduction

*Oplopanax horridus* (Sm.) Torr. & A. Gray ex Miq., commonly known as Devil’s club, is a botanical exclusively originated from and grown in North America [1,2,3]. *O. horridus* has a long and well-known history of medicinal used for rheumatoid arthritis, autoimmune conditions, eczema, type II diabetes, external infections and internal infections, and Devil’s club extracts are marketed in North America, where it eaten as a respiratory stimulant and expectorant [4,5,6]. Traditionally, the native American tribes of the Pacific also used Devil’s club as a spiritual stimulant when Shamans held their ceremonial religious practices [4]. Nowadays, the fresh juice from the root bark of Devil’s club is mostly used for respiratory diseases [5,6]. The volatile oil from the root bark of this plant is regarded as responsible for some clinical uses of the title plant [5,7].

During the past decade, distillation had been used to extract essential oils from different part of *O. horridus* [7,8]. However, distillation has some disadvantages such as thermal degradation, low extraction efficacy and the fact it is labor consuming [9]. SFE can extract volatile oils at lower temperatures, hence avoiding thermal degradation and the use of toxic solvents [10,11]. Therefore, SFE using carbon dioxide as solvent was employed and the parameters, including system pressure, extraction temperature and static extraction time were optimized to obtain higher yields of volatile oil from the root bark of the title plant.

Volatile oils from natural medicines are very complicated, so some volatile compounds have never been identified before by GC/MS. Hereby, the qualitative analyses of those volatile compounds are mostly focused on their determination and identification, which sometimes provides uncertain results without standards [12]. To date, no simple compounds had been isolated and purified from the essential oil of this herb. Therefore, it is necessary to get some overall knowledge about the volatile compounds in the essential oil. It is especially critical and vital to elucidate the chemical structures of the volatile compound unambiguously by multiple spectroscopic analysis methods, and not only GC/MS detection and identification. For further study, modern chromatographic techniques were used to purify simple compounds from the essential oil of *O. horridus*, and various spectroscopic methods were then employed to identify their structures.

**Figure 1:**
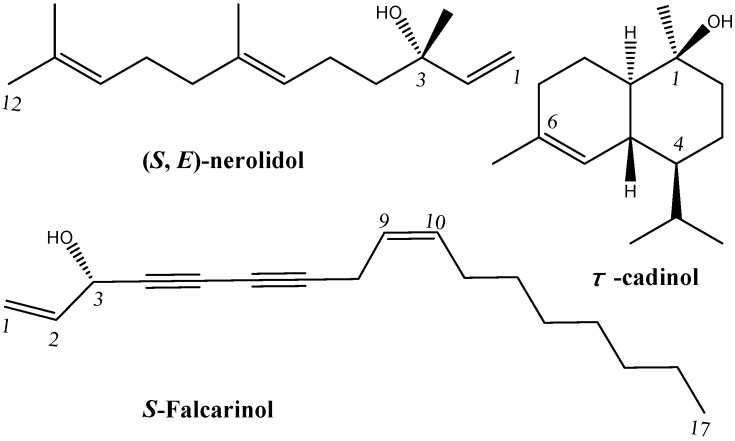
Chemical structures of the isolated volatile compounds.

In this study, the supercritical fluid extraction method was employed first to extract the volatile oil from the root bark of *O. horridus*, which was subsequently analyzed by GC/MS. Forty-eight volatile compounds were identified by GC/MS analysis, including (*S*,*E*)-nerolidol (52.5%), τ-cadinol (21.6%) and *S*-falcarinol (3.6%). Accordingly, the volatile oil (100 g) was subjected to chromatographic separation and purification. As a result, the three compounds, (*E*)-nerolidol (2 g), τ-cadinol (62 mg) and *S*-falcarinol (21 mg) (Figure 1), were isolated and purified for the first time from the volatile oil, the structures of which were unambiguously elucidated by detailed spectroscopic analysis, including 1D- and 2D-NMR techniques.

## 2. Results and Discussion

### 2.1. Development of SFE

SFE was performed using the root bark of *O. horridus* (10 g, dried powder, 80 mesh) as sample which contained all of the volatiles. The pressure, which usually has a significant effect on the extraction efficiency, was set at 320, 360 and 400 bar, respectively. The selected pressure was enough to extract volatile oils from herbs [12]. Actually, the upper pressure limit could be set at approximately 660 bar, but it is not safe to operate the machine at such a high pressure. Other parameters, including vessel temperature (35, 40 and 45 °C) and static extraction time (2.5, 3 and 3.5 h), were optimized using a univariate approach. The dynamic extraction time was set at 0.5 h as a default value. Total peak areas of (*S*,*E*)-nerolidol, τ-cadinol and *S*-falcarinol were selected as the markers for evaluation of extraction efficiency. For SFE conditions, the investigated levels of selected factors including 320 bar, 360 bar and 400 bar as extraction pressure, 35 °C, 40 °C and 45 °C as vessel temperature and 2.5 h, 3 h and 3.5 h as static extraction time were defined as P1, P2, and P3, respectively. To determine one of the parameters, the others were set at the selected default values (temperature, 40 °C; vessel pressure, 360 bar (*ca.* 5,220 psi); static extraction time, 3 h).

**Figure 2:**
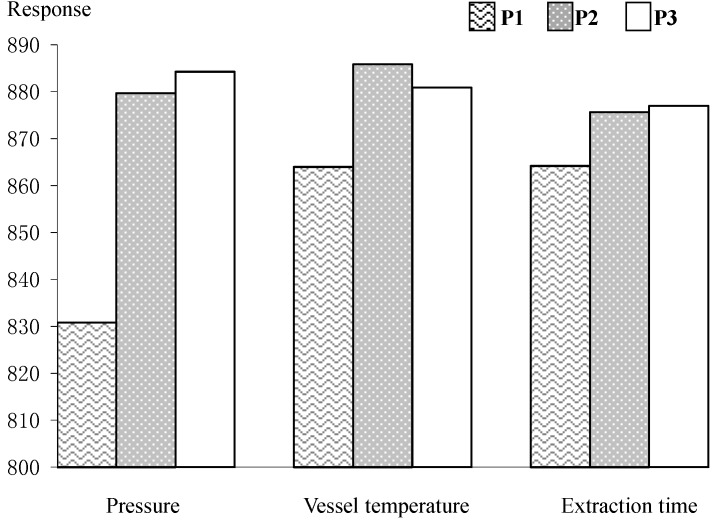
Influence of system pressure, vessel temperature and static extraction time on SFE of three investigated compounds in the volatile oil from the roots bark of *O. horridus*. Conditions: The investigated levels of selected factors, 320 bar, 360 bar, and 400 bar as extraction pressure, 35 °C, 40 °C and 45 °C as temperature and 2.5 h, 3 h and 3.5 h as static extraction time were defined as P1, P2, and P3, respectively. To determine one of the parameters, the others were set at the selected default values (temperature, 40 °C; vessel pressure, 360 bar (*ca.* 5220 psi); static extraction time, 3 h).

The results are shown in Figure 2, which provides the histogram of total integrated peak area of the three volatiles under the selected SFE conditions. It could be easily observed that SFE had higher extraction efficiency when the system pressure, vessel temperature and extraction time were set 400 bar, 40 °C and 3.5 h, respectively. However, it showed no significant difference with the extraction pressure between 360 bar and 400 bar. Considering the operation under very high pressure, we selected 360 bar as the extraction pressure. Similarly, it could also be concluded that there was no obvious difference with the extraction time between 3 h and 3.5 h. Therefore, 3 h were chosen as the extraction time. The recovery of the SFE procedures was determined by performing consecutive supercritical fluid extractions on the same sample under optimized SFE conditions, until no investigated volatiles were detected by GC/MS. The recovery was calculated according to the total amount of individual investigated compounds, which was more than 90% for the first extraction. Considering the results of optimization and recovery experiments, the optimized conditions of the SFE method were: pressure, 360 bar; vessel temperature, 40 °C; static extraction time, 3 h; two extraction cycles.

### 2.2. Validation of the Developed Method

The overall intra- and inter-day variations (RSDs) of the three analytes were between 1.7%~3.3%, respectively (Table 1). The developed method showed good accuracy with overall recovery of 95.3%–106.2% for the three volatiles (Table 1). The extraction recovery determined for the selected compounds was shown to be precise and reproducible (Table 1). The extraction recoveries were between 97.6%~103.6%. The results indicated that this GC/MS coupled with SFE method was precise and accurate for qualitative analysis of volatile components.

**Table 1:** Precision and recovery of investigated volatiles in roots bark of *O. horridus*.

Analytes	Intra-day (*n* = 6) RSD%	Inter-day (*n* = 6) RSD%	Repeatability (*n* = 3) RSD%			Recovery (*n* = 3) (%)
			HR *	MR *	LR *	
**(*S*, *E*)-Nerolidol**	2.9	2.1	4.0	2.3	2.2	97.6
**τ-Cadinol**	1.8	2.2	3.8	3.7	2.6	103.6
***S*-Falcarinol**	1.7	3.3	4.2	2.5	3.9	99.1

Note: * HR, MR and LR represent the levels NMR of 20 g, 10 g and 5 g roots bark powder of *O. horridus*, respectively.

### 2.3. GC/MS Determination and Identification

**Figure 3:**
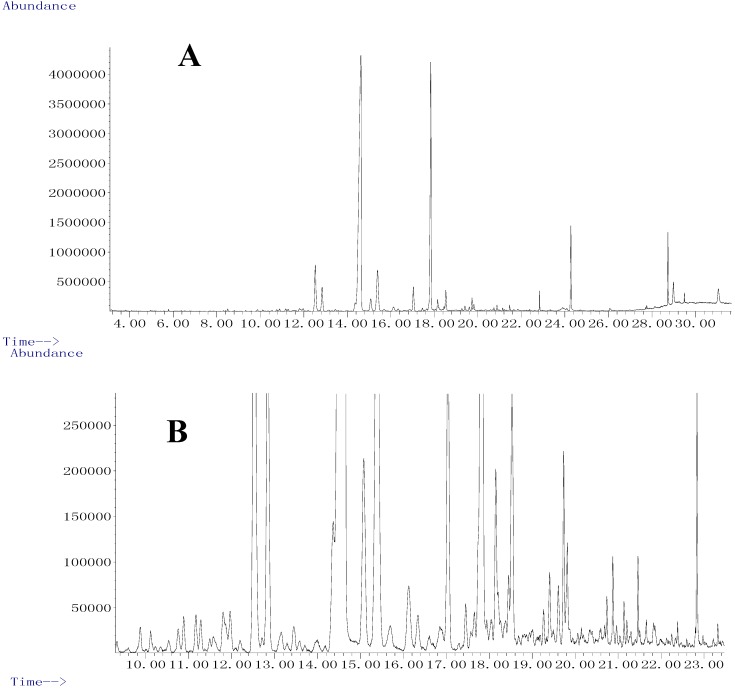
The total ion current profiles of essential oil from *O. horridus*; original profile (**A**) and enlarged profile from 9 to 25 min (**B**).

Since it is preferred to isolate and purify simple compounds from the essential oil, about 10.0 kg of material were used for the essential oil extraction to afford 115 g of oil with a yield of 1.15%, which was much more than the yield of 0.22% when the oil was extracted by the steam distillation method [7]. Most of the slight yellowish oil was subjected to the separation studies, while some 20 mg samples were used for GC/MS analysis (Figure 3A,B).

The chemical composition of essential oil from *O. horridus* is displayed in Table 2. Among all the detectable constituents, (*S*,*E*)-nerolidol (52.5%) was the compound with highest content in the volatile oil, followed by τ-cadinol (21.6%) and bicyclogermacrene (4.5%). (*S*,*E*)-Nerolidol and τ-cadinol had been purified as simple compounds from the essential oil and their structures were identified by additional spectroscopic techniques. By comparison with the MS data from the Agilent Chemstation the library, one more polyyne, *S*-falcarinol (3.6%), was also detected, and was also obtained from the essential oil by chromatographic separation. Except for some very common volatile compounds e.g., α-pinene, β-phellandrene, linalool, 1,3,5-undecatriene and 1,3,5,8-undecatetraene, most of the constituents identified in the essential oil were sesquiterpenes and oxygenated sesquiterpenes. Some minor constituents were not identified by GC/MS. To the best of our knowledge, this is the first time that GC/MS coupled with SFE was used to investigate the essential oil components from the root bark of *O. horridus* with the highest yield.

**Table 2:** The chemical composition of essential oil from the roots bark of *O. horridus*.

Compound	RI HP-5 ^a^	Molecular Formula	Relative Content (%)
Pentanol	826	C_5_H_12_O	0.02
Hexanol	834	C_6_H_14_O	0.05
Heptanal	856	C_7_H_14_O	0.01
Heptanol	862	C_7_H_16_O	0.18
Octanol	868	C_8_H_16_O	0.21
Benzaldehyde	876	C_7_H_6_O	0.06
2-Octenal	880	C_8_H_14_O	0.13
2-Methylpentenal	892	C_6_H_12_O	0.07
α-Pinene	931	C_10_H_16_	0.18
β-Phellandrene	986	C_10_H_16_	0.06
Linalool	1087	C_10_H_18_O	0.62
1,3,5-Undecatriene	1126	C_11_H_18_	0.68
1,3,5,8-Undecatetraene	1189	C_11_H_20_	0.21
α-Ylangene	1263	C_15_H_24_	0.08
Aromadendrene	1312	C_15_H_24_	0.06
α-Zingiberene	1389	C_15_H_24_	0.28
β-Caryophyllene	1411	C_15_H_24_	0.16
(*E*)-α-Bergamotene	1421	C_15_H_24_	0.04
α-Copaene	1433	C_15_H_24_	1.01
α-Humulene	1438	C_15_H_24_	0.43
α-Cadinene	1442	C_15_H_24_	0.06
Germacrene A	1454	C_15_H_24_	0.22
(*E*)-β-Farnesene	1462	C_15_H_24_	0.11
γ-Muurolene	1482	C_15_H_24_	0.07
Germacrene D	1496	C_15_H_24_	0.34
ar-Curcumene	1510	C_15_H_22_	0.04
β-Sesquiphellandrene	1521	C_15_H_24_	0.48
β-Elemene	1533	C_15_H_24_	1.04
α-Muurolene	1542	C_15_H_24_	0.53
allo-Aromadendrene	1544	C_15_H_24_	0.49
γ -Cadinene	1551	C_15_H_24_	1.02
α-Farnesene	1557	C_15_H_24_	0.35
1,10-Di-epi-cubenol	1562	C_15_H_24_O	0.25
δ-Cadinene	1568	C_15_H_24_	0.28
Bicyclogermacrene	1572	C_15_H_24_	4.51
Ishwarane	1577	C_15_H_24_	0.38
Germacrene B	1577	C_15_H_24_	1.14
(*S*,*E*)-Nerolidol	1589	C_15_H_26_O	52.52
Spathulenol	1592	C_15_H_24_O	0.78
Germacrene D-4-ol	1610	C_15_H_26_O	0.63
Gleenol	1621	C_15_H_26_O	0.47
Guaiol	1630	C_15_H_26_O	0.52
Endo-1-bourbonanol	1638	C_15_H_26_O	0.54
τ-Cadinol	1644	C_15_H_26_O	21.62
τ-Muurolol	1655	C_15_H_26_O	1.03
*S*-Falcarinol	1660	C_17_H_24_O	3.63
α-Eudesmol	1670	C_15_H_26_O	0.09
Bulnesol	1677	C_15_H_26_O	0.11

Note: ^a^ RI, retention indices referred to C_8_–C_26_ n-alkanes on HP-5MS capillary column.

## 3. Experimental Section

### 3.1. General Procedures

Optical rotations were recorded on a Model 341 polarimeter (PerkinElmer, Waltham, MA, USA). UV spectra were measured by a DU 640 spectrophotometer (Beckman Coulter, Brea, CA, USA). IR spectra were recorded with a PerkinElmer Spectrum 100 FT-IR spectrometer with KBr pellets. The ^1^H-, ^13^C-, and 2D-NMR spectra (δ in ppm, J in Hz) were recorded on a Bruker AV-500 spectrometer with tetramethylsilane (TMS) as an internal standard (Bruker, Karlsruhe, Germany). ESI-MS and HR-ESI-MS measurements were carried out on an Agilent 1100 series LC/MSD Trap VL mass spectrometer (Agilent Technologies, Palo Alto, CA, USA). Silica gel (200–300 mesh) (Qingdao Haiyang Chemical Co. Ltd, Qingdao, China) and Alltech Reversed-phase C_18_ (RP-C_18_) silica gel (40–63 µm) (Alltech, Columbia, MD, USA) were used for column chromatography (CC). Precoated silica gel GF_254_ plates (Qingdao Haiyang Chemical Co. Ltd., Qingdao, China) were used for TLC. Supercritical fluid extraction was manipulated on a supercritical fluid extractor (SFT-250, Supercritical Fluid Technologies, Inc., Newark, NJ, USA). Analytical HPLC was performed on an Agilent 1200 liquid chromatography with a Luna RP-C_18_ column (250 mm × 4.6 mm inside diameter (I.D.), 5µm, Phenomenex, Torrence, CA, USA). Preparative HPLC was carried out with an Agilent 1200 liquid chromatograph with a Phenomenex Luna RP-C_18_ column (250 mm × 22 mm I.D., 5 µm). HPLC-grade methanol and Acetonitrile were the products of Merck (Darmstadt, Germany). The deionized water used for HPLC was purified by a Milli-Q purification system (Millipore, Bedford, MA, USA). All the analytical grade organic solvents were purchased from Sinopharm Chemical Reagent Co., Ltd (Shanghai, China). The glassware used for chemical experiments were bought from Buchi (Flawil, Switzerland). The glass columns packed with separation materials were purchased from the Xiamei Company (Shanghai, China).

### 3.2. Plant Material

The air-dried roots bark of *O. horridus* was purchased from Pacific Botanicals Co. Ltd. (Chicago, IL, USA) and authenticated by one of the authors (C.-Z. Wang) in March, 2012. A voucher specimen (Lot: OHR-20120926-1) has been deposited in the Institute of Clinical Pharmacology, Central South University, Hunan Province, China.

### 3.3. Supercritical Fluid Extraction of Volatiles from O. horridus

The volatile oil from the roots bark of *O. horridus* was extracted by the SFT-250 SFE/SFR system. The settlement of parameters was selected on the basis of pressure, temperature, static time (modifier selected according to the preliminary investigation), which had crucial efficiency on the volatile oil extraction of SFE. The primary parameters for volatile oil extraction by SFE were selected according to practical experience and literature data [13]. Supercritical CO_2_ was used as the extraction agent. Finally, the extraction procedure was operated under the optimized conditions: the vessel pressure was set at 360 bar, while the vessel temperature and static time were settled at 40 °C and 3 h for the variable levels, respectively. The SFE coupled with the 5000 mL extraction vessel was used for the preparation of volatile oil from 10.0 kg powder of *O. horridus* under the selected conditions. The extracted volatile oil (115 g) was collected in a 500 mL glass bottle, which was used for chemical isolation and GC/MS analysis.

### 3.4. GC/MS Analysis

GC/MS was performed on an Agilent 6890 gas chromatography instrument coupled with an Agilent 5973 mass spectrometer (Agilent Technologies). An HP-5MS capillary column (30 m × 0.25 mm, I.D.) coated with 0.25 μm film 5% phenyl methyl siloxane was used for separation. The column temperature was set at 100 °C and held for 2 min, then programmed at 8 °C·min^−1^ to 160 °C and held for 10 min, then at 10 °C·min^−1^ to 200 °C and held for 6 min, continued at 20 °C·min^−1^ to 320 °C and held for 3 min. Split injection (1 μL) with a split ratio of 1:50 was applied. High purity helium was used as carrier gas with flow rate of 1.0 mL·min^−1^. The mass spectrometer was operated in electron-impact (EI) mode, the scan range was 40–550 amu, the ionization energy was 70 eV and the scan rate was 2.89 s per scan. The inlet, ionization source temperature were 250 and 285 °C, respectively.

### 3.5. Method Validation

To validate the GC/MS analysis coupled with SFE experiments developed in the current study, Intra- and inter-day variations were used to determine the precision of the method. The known concentrations of three isolated volatiles were tested. For intra-day variability tests, the standard solutions were analyzed six times within one day, while for inter-day variability tests, the samples were examined in duplicate on three consecutive days. Variations were expressed as the relative standard deviations (RSD). The recovery was determined to evaluate accuracy of the method. A known amount of individual isolated compounds were added to 10 g of the root bark of *O. horridus*. The mixture was then extracted and analyzed using the methods developed above. Three replicates were performed for the test. The extract was transferred into a 5 mL volumetric flask which was made up to its volume with ethanol and filtered through a 0.45 μm filter before analysis. The integrated peak area of each volatile was subsequently calculated. The recovery was calculated as follow: Recovery (%) = 100 × (peak area found − original peak area)/peak area spiked.

### 3.6. Chemical Isolation of Volatile Compounds

The extracted volatile oil (100 g) was then subjected to silica gel (200–300 mesh) column chromatography (CC) and eluted with gradient mixtures of petroleum ether (boiling point: 30–60 °C) and ether (100:0 to 0:100) to afford nine fractions (OP1-OP9). Fraction OP5 (24 g) was then subjected to silica gel (200–300 mesh) CC, eluting with petroleum ether–ether (50:1, 10:1 and 5:1), to give six subfractions (OP5a–OP5f). Subfraction OP4d (6 g) was chromatographed by Prep-HPLC (MeOH–H_2_O, 82:18) to afford (*S*,*E*)-nerolidol (2 g) and τ-cadinol (62 mg). Fraction OP7 (5 g) was then subjected to silica gel (200–300 mesh) CC eluting with petroleum ether–ether (20:1, 10:1 and 4:1), to give six subfractions (OP7a–OP7f). Subfraction OP7e (1 g) was chromatographed by Prep-HPLC (MeOH–H_2_O, 80:20) to yield *S*-falcarinol (21 mg). The flow rate was set at 10 mL·min^−1^ for Prep-HPLC. The characterization data was recorded in the supplementary materials.

## 4. Conclusions

A GC/MS coupled with SFE method was first employed to investigate the volatile oil from the root bark of *O. horridus*. Forty-eight volatile compounds were identified by GC/MS analysis, including three high content compounds, (*S*,*E*)-nerolidol, τ-cadinol and *S*-falcarinol, which were isolated and purified for the first time from the volatile oil, and their structures were unambiguously elucidated by detailed spectroscopic analysis, including NMR techniques. The bioactivities of isolated volatiles needs further study.

## Supplementary Materials

Supplementary materials can be accessed at: http://www.mdpi.com/1420-3049/19/12/19708/s1.
