# Supplementary material for: Unstable Simple Volatiles and Gas Chromatography-Tandem Mass Spectrometry Analysis of Essential Oil from the Roots Bark of Oplopanax Horridus Extracted by Supercritical Fluid Extraction
Source: Molecules. 2014 Nov 27;19(12):19708–17. doi: 10.3390/molecules191219708 (PMC6271617; doi:10.3390/molecules191219708)
Supplement: Supplementary file 1 [file molecules-19-19708-s001.pdf]

Supplementary Materials

Structure Elucidation of Isolated Volatiles

(*S*,*E*)-Nerolidol was obtained as light viridescent oil. The molecular formula of (*S*,*E*)-Nerolidol was determined to be $C_{15}H_{26}O$ according to the electrospray ionization (ESI)-MS spectra (*m/z* 245 $[M+Na]^+$) as well as ^{1}H- and ^{13}C-NMR spectrum. The IR absorptions (3420, 3128 and 1620 cm^{-1}) indicated the presence of hydroxyl and C=C bond. In the ^{1}H-NMR spectra of (*S,E*)-Nerolidol, five olefinic protons signals at δ_H 5.92 (1H, ddd, *J* = 17.4, 10.0, 5.5 Hz), 5.22 (1H, dd, *J* = 17.5, 1.0 Hz), 5.14 (1H, tq, *J* = 7.0, 1.0 Hz), 5.10 (1H, m) and 5.07 (1H, dd, *J* = 10.0, 1.0 Hz) and four methyl group at δ_H 1.68 (3H, s), 1.60 (6H, s) and 1.34 (3H, s) could be unambiguously observed. The ^{13}C-NMR spectra revealed the presence of 15 carbons, including six olefinic carbons at δ_C 145.0, 135.5, 131.3, 124.9, 124.2 and 111.7, four methyl carbons (δ_C 27.8, 26.6, 17.6 and 16.0), one oxygen-bearing *sp*^{3} carbons at at δ_C 73.5, and the remaining four methylene groups at δ_C 42.0, 39.7, 25.6 and 22.7. Based on the coupling constant values of the five olefinic protons, three independent vinyl groups were confirmed in the structure. On the basis of these structural determinations, GC/MS analysis and comparison the NMR spectrum data with the reported compounds, the structure of (*S*,*E*)-Nerolidol was identified as (*S*,*E*)-3,7,11-trimethyldodeca-1,6,10-trien-3-ol [1,2].

(*S*,*E*)-nerolidol Slight viridescent oil. ESI-MS *m/z*: 245 $[M+Na]^+$. $[\alpha]_D^{25}$ +153.0° (c = 0.9, CH_3OH); UV λ_{max}(CH_3OH): 220, 206 and 194. IR (KBr) ν_{max}: 3420, 3128, 1620, 1115 and 824 cm^{-1}. ^{1}H-NMR ($CDCl_3$, 500M): δ_H 5.92 (1H, ddd, *J* = 17.4, 10.0, 5.5 Hz, H-1b), 5.22 (1H, dd, *J* = 17.5, 1.0 Hz, H-2), 5.14 (1H, tq, *J* = 7.0, 1.0 Hz, H-10), 5.10 (1H, m, H-7), 5.07 (1H, dd, *J* = 10.0, 1.0 Hz, 1a), 2.08–1.96 (6H, m, H-5, H-8, H-9), 1.68 (3H, s, H-12), 1.60 (6H, s, H-13, -14), 1.58 (2H, m, H-4), 1.35 (3H, s, H-15). ^{13}C-NMR ($CDCl_3$, 125M): δ_C 145.0 (C-2), 135.5 (C-7), 131.3 (C-11), 124.9 (C-16), 124.2 (C-10), 111.7 (C-1), 73.5 (C-3), 42.0 (C-4), 39.7 (C-8), 27.8 (C-15), 26.6 (C-9), 25.6 (C-12), 22.7 (C-5), 17.6 (C-13), 16.0 (C-14). GC/MS (EI) *m/z* (%): 222 (12), 204 (41), 136 (52), 107 (100), 93 (26), 81 (16), 69 (10).

τ-cadinol was yielded as colorless oil. Its molecular formula, $C_{15}H_{26}O$, was calculated on the basis of its molecular ion at *m/z* 222 $[M-H]^-$ in ESI-MS spectra together with the ^{1}H- and ^{13}C-NMR spectrum. A hydroxyl and vinyl characteristic absorptions could be noted in the IR spectra at 3510, 3120 and 1558 cm^{-1}. Only one olefinic protons signal was found in the ^{1}H-NMR spectra of *τ*-cadinol at δ_H 5.54 (1H, br.d, *J* = 3.0 Hz), as well as four methyl protons signals at δ_H 1.67 (3H, s), 1.22 (3H, s), 0.92 (3H, d, *J* = 7.5 Hz) and 0.89 (3H, d, *J* = 7.1 Hz) noted. Analysis of the ^{13}C-NMR spectra, 15 carbons were elucidated in the molecular structure, containing two olefinic carbons at δ_C 134.3. and 126.6, four methyl carbons (δ_C 28.4, 26.6, 21.4 and 15.2), one oxygen-bearing *sp*^{3} carbons at at δ_C 70.8, and the remaining eight *sp*^{3} carbons at δ_C 47.9, 46.7, 37.7, 31.0, 23.7, 22.7, 21.4 and 19.8. *τ*-cadinol was applied for GC-MS analysis with these structural elucidation and comparison the NMR spectrum data with literatures, which was characterized as (1*S*,4*S*,4a*R*,8a*R*)-4-isopropyl-1,6-dimethyl-1,2,3,4,4a,7,8,8a-octahydronaphthalen-1-ol [1,3].

τ-cadinol Colorless oil. ESI-MS *m/z*: 221 $[M-H]^-$. $[\alpha]_D^{25}$ +62.0° (*c* = 0.7, CH_3OH); UV λ_{max}(CH_3OH): 205 and 192. IR (KBr) ν_{max}: 3510, 3120, 1558, 1014 and 925 cm^{-1}. ^{1}H-NMR ($CDCl_3$, 500M): δ_H 5.54 (1H, br.d, *J* = 3.0 Hz, H-5), 2.09–1.94 (2H, m, H-3), 1.96 (1H, m, H-6), 1.93 (1H, m, H-2a), 1.75 (1H, m, H-9a), 1.67 (3H, s, H-11), 1.46 (1H, m, H-8a), 1.41 (1H, m, H-9b), 1.35 (1H, m, H-2a), 1.22 (3H, s, H-15), 1.08 (1H, m, H-7a), 0.92 (3H, d, *J* = 7.5 Hz, H-13), 0.80 (3H, d, *J* = 7.1 Hz,

H-14); ^{13}C-NMR ($CDCl_3$, 125M): δ_C 134.3 (C-4), 126.6 (C-5), 70.8 (C-10), 47.9 (C-1), 46.7 (C-7), 40.4 (C-9), 37.7 (C-6), 31.0 (C-3), 28.4 (C-15), 26.6 (C-12), 23.7 (C-11), 22.7 (C-2), 21.4 (C-13), 19.8 (C-8), 15.2 (C-15). GC/MS (EI) *m/z* (%): 222 (21), 204 (45), 148 (42), 105 (100), 91 (34), 79 (22), 63 (16).

S-Falcarinol was purified as light yellowish oil. The ESI-MS of *S*-Falcarinol displayed a molecular ion at *m/z* 267 $[M+Na]^+$, which suggested the molecular formula of *S*-Falcarinol was determined to be $C_{17}H_{24}O$ from a combination of ^{1}H- and ^{13}C-NMR spectrum data. The UV (285, 268, 260 and 250 nm) and IR absorptions (2252 and 1682 cm^{-1}) implied the existence of two C≡C bonds [4]. Analysis of the ^{1}H-NMR spectra of *S*-Falcarinol, five olefinic protons signals at δ_H 5.93 (ddd, *J* = 17.4, 10.0, 5.5 Hz), 5.64 (ddt, *J* = 10.6, 7.5, 1.0 Hz), 5.51 (ddt, *J* = 10.6, 8.2, 1.0 Hz), 5.48 (dt, *J* = 17.4, 1.0 Hz) and 5.25 (dt, *J* = 10.0, 1.0 Hz), an oxygen-bearing sp^3 carbon proton at δ_H 4.95 (1H, d, *J* = 6.0 Hz), seven methylene groups at δ_H 2.43 (2H, d, *J* = 6.7 Hz), 2.10 (2H, tq, *J* = 7.1, 1.5 Hz), 1.38 (2H, m) and 1.28 (8H, m) and one methyl group at δ_H 0.89 (3H, t, *J* = 7.2 Hz) could be observed.

The ^{13}C-NMR spectra provided 17 carbon rods signals, including four olefinic carbons at δ_C 135.3, 132.4, 124.6 and 117.2, seven methylenes carbons (δ_C 31.7, 29.2, 29.1, 29.1, 27.6, 22.6 and 18.3), one methyl (δ_C 9.4), four unprotonated acetylenic carbons and one oxygen-bearing sp^3 carbons at δ_C 63.6. The vicinal coupling constant between H-9 and H-10 ($J_{9,10}$ = 10.7 Hz) decided that the geometry of the double bond between C-9 and C-10 to be *cis* as the double bond fixed to be ***Z***. From the above analysis and comparison the NMR data with known compound, the structure of (*S*)-falcarindiol was elucidated as (*S,Z*)-heptadeca-1,9-dien-4,6-diyne-3-ol [5].

(S)-falcarindiol yellowish oil; $[\alpha]_D^{25}$ +173.5° (*c* = 0.67, $CHCl_3$); UV ($CHCl_3$) λ_{max} (log ξ): 220 (0.78), 250 (0.92), 260 (1.59), 268 (1.78) and 285 (2.13) nm; IR (KBr) ν_{max}: 3352, 3018, 2935, 2852, 2252, 1682, 1468, 1022, 936 and 876 cm^{-1}; ^{1}H-NMR(500 MHz, $CDCl_3$): δ_H 5.93 (1H, ddd, *J* = 17.5, 10.0, 6.0 Hz, H-2), 5.64 (1H, dd, *J* = 17.5, 1.5 Hz, H-1b), 5.51 (1H, ddt, *J* = 11.5, 5.0, 1.5 Hz, H-9), 5.48 (1H, ddt, *J* = 11.5, 7.5, 1.0 Hz, H-10), 5.25 (1H, d, *J* = 10.0 Hz, H-1a), 4.95 (1H, brd, *J* = 5.0 Hz, H-3), 2.43 (1H, d, *J* = 6.7 Hz, H-8), 2.10 (2H, q, *J* = 7.0, H-11), 1.38 (2H, m, H-12), 1.28 (8H, m, H-13, H-14, H-15, H-16), 0.89 (3H, t, *J* = 7.0 Hz, H-17). ^{13}C-NMR (125 MHz, $CDCl_3$): δc 9.4 (C-17), 18.3 (C-8), 22.6 (C-15), 27.6 (C-11), 29.0 (C-12), 29.1 (C-14), 29.2(C-13), 31.7 (C-16), 63.6 (C-3), 68.8 (C-6), 70.7 (C-5), 78.5 (C-4), 77.8 (C-7), 117.2 (C-1), 124.6 (C-10), 132.4 (C-9), 135.3 (C-2). GC/MS (EI) *m/z* (%): 244 (18), 202 (100), 118 (46), 105 (45), 79 (21).

References

1. Sun, S.; Du, G.J.; Qi, L.W.; Williams, S.; Wang, C.Z.; Yuan, C.S. Hydrophobic constituents and their potential anticancer activities from Devil's club (*Oplopanax horridus* Miq.). *J. Ethnopharmacol.* **2010**, *132*, 280–285.
2. Dittmer, D.C.; Discordia, R.P.; Zhang, Y.Z.; Murphy, C.K.; Kumar, A.; Pepito, A.S.; Wang, Y.S. A tellurium transposition route to allylic alcohols-overcoming some limitations of the sharpless katsuki asymmetric epoxidation. *J. Org. Chem.* **1993**, *58*, 718–731.
3. Appendino, G.; Jakupovic, J.I.; Jakupovic, S. Sesquiterpenoids from *Pallenis spinosa*. *Phytochemistry* **1997**, *46*, 1039–1043.

4. Crosby, D.G.; Aharonson, N. The structure of carotatoxin, a natural toxicant from carrot. *Tetrahedron* **1967**, *23*, 465–472.
5. Li, Z.Y.; Wang, M.; Bian, Q.H.; Zheng,B.; Mao, J.Y.; Li, S.N.; Liu, S.Z.; Wang, M.A.; Zhong, J.C.; Guo, H.C. Highly enantioselective addition of trimethylsilylacetylene to aldehydes catalyzed by a zinc-amino-alcohol complex. *Chem. Eur. J.* **2011**, *17*, 5782–5786.
